# Conditional deletion of E11/Podoplanin in bone protects against ovariectomy-induced increases in osteoclast formation and activity

**DOI:** 10.1042/BSR20190329

**Published:** 2020-01-10

**Authors:** Katherine A. Staines, Mark Hopkinson, Scott Dillon, Louise A. Stephen, Robert Fleming, Antonia Sophocleous, David J. Buttle, Andrew A. Pitsillides, Colin Farquharson

**Affiliations:** 1 School of Applied Sciences, Edinburgh Napier University, Sighthill Campus, Edinburgh, U.K.; 2 Comparative Biomedical Sciences, Royal Veterinary College, Royal College Street, London, U.K.; 3 Roslin Institute and R(D)SVS, The University of Edinburgh, Easter Bush, Midlothian, U.K.; 4 Department of Life Sciences, School of Sciences, European University Cyprus, Cyprus, Nicosia; 5 Department of Infection, Immunity and Cardiovascular Disease, University of Sheffield, Sheffield, U.K.

**Keywords:** bone, E11/podoplanin, osteoclast, osteocyte, osteoporosis

## Abstract

E11/Podoplanin (Pdpn) is implicated in early osteocytogenesis and the formation of osteocyte dendrites. This dendritic network is critical for bone modelling/remodelling, through the production of receptor activator of nuclear factor κ B (RANK)-ligand (RANKL). Despite this, the role of Pdpn in the control of bone remodelling is yet to be established *in vivo*. Here we utilised bone-specific *Pdpn* conditional knockout mice (cKO) to examine the role of Pdpn in the bone loss associated with ovariectomy (OVX). MicroCT revealed that Pdpn deletion had no significant effect on OVX-induced changes in trabecular microarchitecture. Significant differences between genotypes were observed in the trabecular pattern factor (*P*<0.01) and structure model index (*P*<0.01). Phalloidin staining of F-actin revealed OVX to induce alterations in osteocyte morphology in both wild-type (WT) and cKO mice. Histological analysis revealed an expected significant increase in osteoclast number in WT mice (*P*<0.01, compared with sham). However, cKO mice were protected against such increases in osteoclast number. Consistent with this, serum levels of the bone resorption marker Ctx were significantly increased in WT mice following OVX (*P*<0.05), but were unmodified by OVX in cKO mice. Gene expression of the bone remodelling markers *Rank, Rankl, Opg* and *Sost* were unaffected by Pdpn deletion. Together, our data suggest that an intact osteocyte dendritic network is required for sustaining osteoclast formation and activity in the oestrogen-depleted state, through mechanisms potentially independent of RANKL expression. This work will enable a greater understanding of the role of osteocytes in bone loss induced by oestrogen deprivation.

## Introduction

Throughout life our skeleton is continuously remodeled, a process which is under tight regulation so as to ensure an imbalance in bone formation or bone resorption does not occur. Such imbalance in favour of bone resorption results in pathological bone loss leading to osteopaenia and osteoporosis. These diseases dramatically enhance risk of fracture and as such, are a massive worldwide healthcare burden.

Osteoclasts are responsible for the resorption of mineralised bone through the release of protons for mineral dissolution and enzymes, such as tartrate-resistant acid phosphatase (TRAP) and members of the cathepsin and matrix metalloproteinase (MMP) families for the degradation of the organic bone matrix. [1] Osteoclasts originate from a haemopoietic lineage and their differentiation is stimulated by macrophage colony-stimulating factor (M-CSF), and receptor activator of nuclear factor κ B (RANK)-ligand (RANKL). Despite their opposing functional roles, compelling evidence supports a pivotal role for osteoblasts in the regulation of osteoclast differentiation. [2] More recently, evidence has also implicated a critical role for the osteoblast-derived osteocyte in the regulation of osteoclast differentiation via the production of RANKL. [3,4] Indeed, the selective conditional deletion of RANKL in osteocytes resulted in a marked osteopetrotic phenotype in skeletally mature mice. [4]

Osteocytes are the most abundant cell type of the adult skeleton. It has long been considered that osteocytes are formed by the passive entrapment of redundant osteoblasts by osteoid synthesised by their close neighbours [5,6]. The transition from the cuboidal-like osteoblastic morphology to a dendritic shape characteristic of an osteocyte is however a more active and tightly regulated process than was originally recognised [7,8]. The characteristic dendritic phenotype enables the osteocyte to form a highly connected syncytium with neighbouring osteocytes and surface osteoblasts and osteoclasts, and places the osteocyte at the centre of bone modelling/remodelling regulatory pathways (for reviews see [9,10]).

We, and others, have previously highlighted the requirement for the transmembrane glycoprotein E11/podoplanin (Pdpn) to make the cytoskeletal network modifications that are critical for the formation of dendrites during osteocytogenesis. This role is consistent with its expression by early embedding osteocytes, thus identifying Pdpn as a factor which likely contributes to the vital, early stages of osteocyte differentiation [11]. Furthermore, *Pdpn* expression in osteocytes is increased in response to mechanical strain *in vivo* [12] and the formation of dendritic processes is promoted by both Pdpn overexpression and through its stabilisation by proteasome inhibitors [11,13]. In contrast, the formation of these cytoplasmic processes is abrogated in cells pre-treated with siRNA targeted against *Pdpn* and in mice with a bone-specific ablation of Pdpn [14]. From this evidence, it is reasonable to conclude that Pdpn has an important functional role in the formation of dendritic processes which are a key feature of the differentiating osteocyte. Nonetheless, important gaps remain in our understanding of the essential role played by Pdpn in full osteocyte function; in particular the control of osteoblast and osteoclast actions during the bone remodelling process.

Global deletion of Pdpn is perinatally lethal due to the essential role of Pdpn in lung and epithelial cell function [12]. Therefore, in order to test our working hypothesis that Pdpn drives osteocytogenesis and thus regulates bone remodelling, we have utilised our previously characterised osteocalcin (OC)-Cre driven bone-specific *Pdpn* hypomorphic deletion in mice [14] (conditional knockout mice (cKO)) to compare the effects of ovariectomy (OVX)-induced bone loss in cKO to wild-type (WT) mice.

## Materials and methods

### Animals

Bone-specific *Pdpn* conditional hypomorphic knockout mice (cKO; ∼70% reduction in protein Pdpn expression) were generated as previously described under the control of the OC-Cre promoter [14]. OC-Cre mice (WT) were used as controls. Mice were kept in polypropylene cages, with light/dark 12-h cycles, at 21 ± 2°C, and fed *ad libitum* with maintenance diet (Special Diet Services, Witham, U.K.). All experimental protocols were approved by Roslin Institute’s Animal Users Committee (A650) and experiments were conducted at the Roslin Institute, University of Edinburgh. Animals were maintained in accordance with U.K. Home Office guidelines for the care and use of laboratory animals.

### OVX model of enhanced bone remodelling

At 10 weeks of age, female cKO and WT mice were subjected to either OVX (*n*=6/genotype) or sham operation (*n*=6/genotype), with isoflurane anaesthesia. Tissues were collected 4 weeks post-surgery (14 weeks of age) following exsanguination under terminal anaesthesia. Uterine and body weights were assessed at killing.

### Histological analysis

Right femora were fixed for 24 h in 4% formaldehyde in phosphate buffered saline (PBS) and stored in 70% ethanol. Samples were decalcified in 10% ethylenediaminetetraacetic acid (EDTA) for 21 days at 4°C, with the solution changed every 4–5 days. Samples were washed in PBS, bisected in the sagittal plane, processed to paraffin wax using a Leica (Wetzlar, Germany) ASP300S Tissue Processor, and embedded in wax on the medial cut surface. A Leica rotary microtome was used to cut 3-µm sections which were mounted on Superfrost glass slides (Thermo Fisher Scientific, U.S.A.). Slides were stained with H&E and Goldner’s Trichrome using standard protocols. For TRAP staining 70 mg napthol AS-TR phosphate (Sigma) was dissolved in 250 µl N-N dimethyl formamide (Sigma) and added to 50 ml of 0.2 M sodium acetate buffer pH 5.2. A total of 115 mg sodium tartrate dihydrate (Sigma) and 70 mg fast red salt TR (Sigma) were dissolved into this solution and slides were incubated at 37°C for 2 h. Sections were counterstained in Meyer’s Haematoxylin (Sigma), washed in distilled water and mounted in aqueous mounting medium (Vector Labs). Slides were imaged using a NanoZoomer XR slide scanning system (Hamamatsu Photonics, Japan). Histomorphometry was performed using the BIOQUANT OSTEO (BIOQUANT Image Analysis Corporation, Texas, U.S.A.) software package using the approved ASBMR histomorphometry nomenclature (three sections/mouse; WT sham *n*=6, WT OVX *n*=5, cKO sham *n*=6, cKO OVX *n*=4) [15].

### Phalloidin staining

Wax embedded 10-µm sections were washed in PBS twice for 5 min each, and incubated with 0.1% Triton-X 100 (Sigma–Aldrich) for 30 min and then rinsed with PBS. Slides were then incubated with Alexa Fluor 488–conjugated phalloidin (1:500; Abcam, Cambridge, U.K.) for 1 h. Bone sections were washed in PBS and mounted in ProlongGold (Thermo Fisher Scientific, U.S.A.). Preparations were allowed to dry at room temperature for 12 h. Optical sections were taken using the LSM 880 Airyscan confocal laser scanning microscope using the 488 nm laser excitation and detection settings from 493 to 634 nm. Z-stacks were produced with optimal Nyquist overlap settings using a 63×/1.4na oil immersion lens. Voxel sizes were 0.043 × 0.043 × 0.5 μm in x, y, z planes respectively. A comparable region of interest was analysed for osteocyte parameters in all samples located in the diaphyseal cortical bone. Image stacks were imported into Bitplane Imaris 9.3.0 software and algorithms were created with Imaris FilamentTracer to render and measure dendritic processes. Surface rendering was used for osteocyte cell body measurements.

### Micro-computed tomography analysis

Right tibiae were dissected and frozen at −20°C in dH_2_O until required. The laboratory scans were performed with a 1172 X-Ray Microtomograph (Bruker, Belgium) to evaluate the epiphyseal trabecular bone microarchitecture. High-resolution scans with an isotropic voxel size of 5 µm were acquired (50 kV, 0.5 mm aluminium filter, 0.6° rotation angle, 2 frame averaging). The scans were reconstructed using NRecon software (Bruker) and filters were applied to the images prior to reconstruction to remove artefacts, including beam-hardening and ring artefacts. A 1000-µm section of the metaphysis 250 µm subjacent to the growth plate was taken for analysis of trabecular bone. A 500-µm section of the mid-diaphysis, 3735 µm subjacent to the growth plate, was segmented for analysis of cortical structure. Data were analysed with CtAn software (Bruker). To assess bone mineral density (BMD), BMD phantoms of known calcium hydroxyapatite mineral densities of 0.25 and 0.75 g/cm^3^ were used.

### Serum markers of bone formation and resorption

Blood samples from WT and cKO mice were obtained by cardiac puncture under terminal anaesthesia and serum samples were prepared by centrifugation at 1000×*g* for 10 min at 4°C. Markers of bone formation (P1NP) and bone resorption (Ctx) were quantified by ELISA according to the manufacturer’s instructions (AMS Biotechnology, Abingdon, U.K.).

### RNA extraction and quantitative real-time PCR

Left femurs had their marrow removed by centrifugation before being homogenised in Qiazol reagent (Qiagen) and total RNA was extracted using an RNeasy mini lipid kit (Qiagen), according to the manufacturer’s instructions. Reverse transcription was completed using Superscript II reverse transcriptase (Invitrogen) and gene expression analysis was carried out by RT-qPCR performed on a Stratagene Mx3000P real-time qPCR machine (Stratagene, Santa Clara, U.S.A.) using PrecisionPlus qPCR mastermix with SYBR Green (Primer Design, Southampton, U.K.). Relative gene expression was calculated using the ΔΔ*C*_t_ method [16]. Each cDNA sample was normalised to the housekeeping gene *Gapd*h (sequences not disclosed; Primer Design). Reactions were performed with gene-of-interest primers (5′–3′): Rankl (Tnfsf11) F - CGCCAACATTTGCTTTCGG, R - TTTTAACGACATACACCATCAGC; *Opg* (*Tnfrsf11b*): F - AAATTGGCTGAGTGTTTTGGTG, R - CTGTGTCTCCGTTTTATCCTCTC; *Sost*: F - TGAGAACAACCAGACCATGAAC, R - TCAGGAAGCGGGTGTAGTG (Primer Design) and *Rank* (*Tnfrsf11a*): F - GCGCAACAGTGTTTCCACAG, R - CGCTTGGATCACAGTAAGGCT (Merck).

### Statistical analysis

Data were analysed by one-way analysis of variance, Student’s *t* test or a suitable nonparametric test using GraphPad Prism 6 and following normality checks. Data are expressed as the mean ± S.E.M. Results were analysed blindly. *P*<0.05 was considered to be significant and noted as *; *P*-values of <0.01 and <0.001 were noted as ‘**’ and ‘***’ respectively.

## Results

### OVX of Pdpn cKO and WT mice

**Figure 1:**
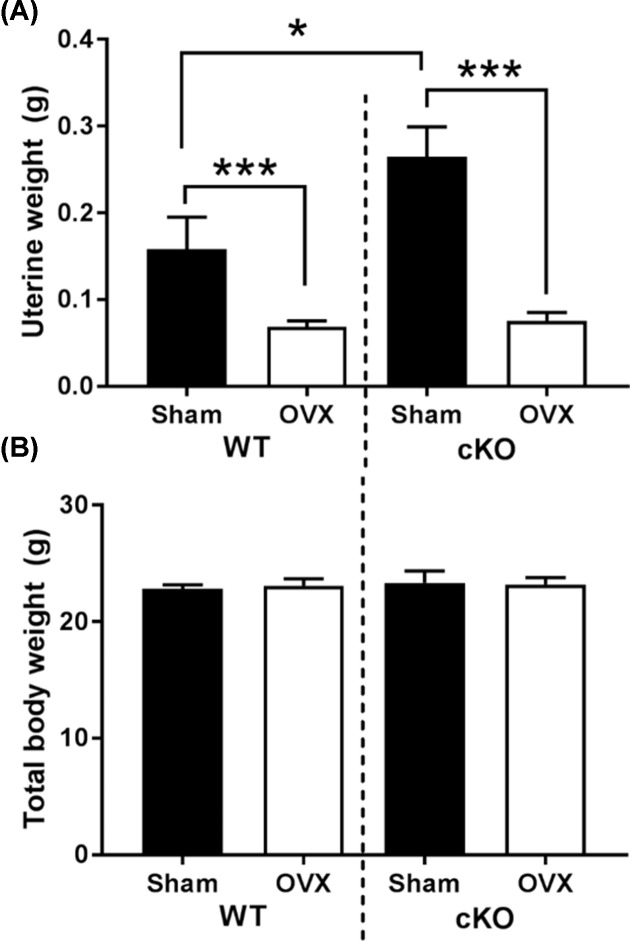
Effects of OVX on WT and Pdpn cKO mice (**A**) Uterine weight (g). (**B**) Total body weight (g). Data are presented as mean ± S.E.M for *n*≥4; *P<0.05; ***P<0.001.

Ten-week old WT and cKO mice were subjected to either OVX or sham surgeries. To evaluate the efficacy of the surgery, uterine weights were examined 4 weeks post-surgery. The effect of OVX was similar in both the cKO and the WT, with both genotypes exhibiting a 30–40% reduction (*P*<0.001) in uterine weight in comparison with the sham operated mice (Figure 1A). Interestingly the uterine weight of the sham operated cKO mice was higher than the equivalent control mice (*P*<0.05). No differences were observed in the total body weight between genotypes and/or surgeries (Figure 1B). These results confirm the success of the OVX and that the hypomorphic deletion of Pdpn does not modify the uterine response to decreased oestrogen.

### Bone microarchitecture changes in response to OVX with Pdpn deletion

**Figure 2:**
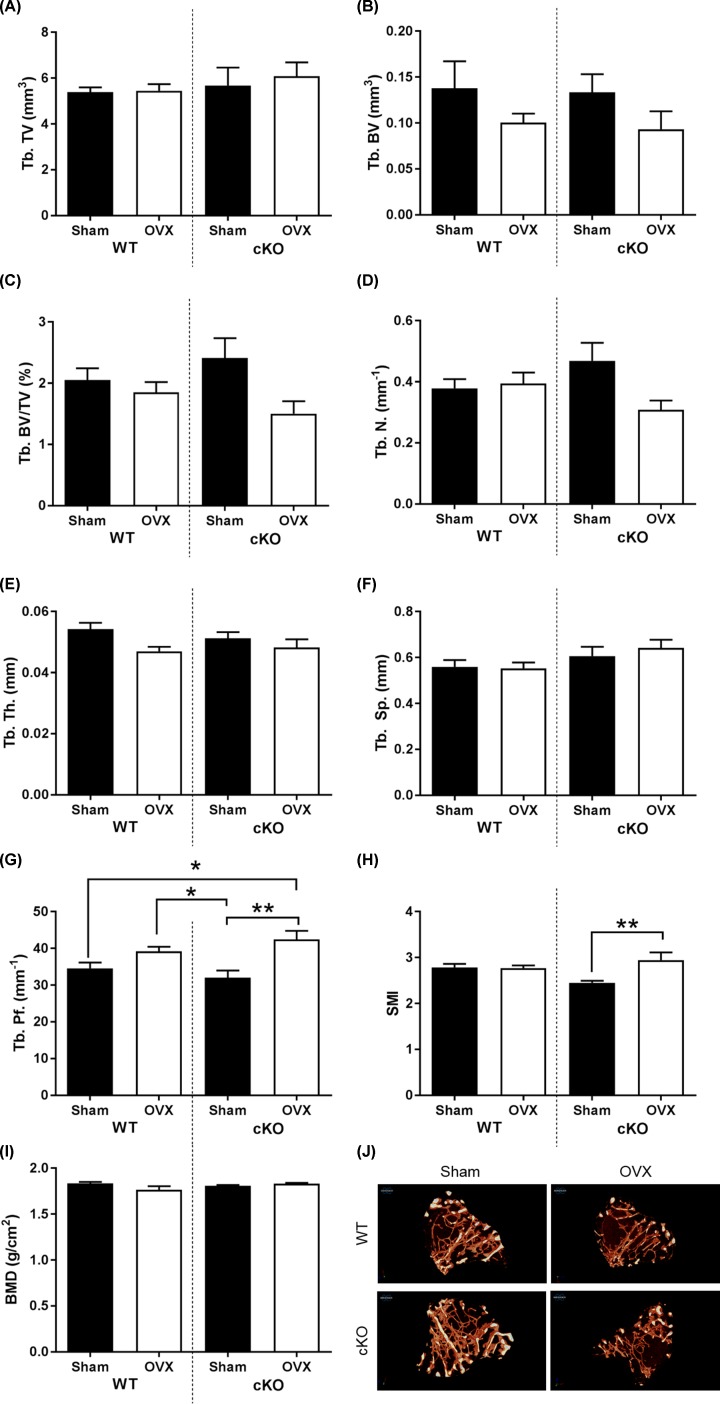
Effects of OVX on WT and Pdpn cKO mouse trabecular bone microarchitecture Micro-CT analysis of tibia trabecular microarchitecture in WT and Pdpn cKO mice in response to OVX (**A**) Tb. TV (Trabecular tissue volume; mm^3^); (**B**) Tb. BV (Trabecular bone volume; mm^3^); (**C**) Tb. BV/TV (Trabecular BV/TV; %); (**D**) Tb. N. (Trabecular Number; mm^−1^); (**E**) Tb. Th. (Trabecular thickness; mm); (**F**) Tb. Sp. (Trabecular Separation; mm); (**G**) Tb. Pf. (Trabecular Pattern Factor; mm^−1^); (**H**) SMI (Structure model index); (**I**) BMD (g/cm^3^). (**J**) Representative images. Data are presented as mean ± S.E.M for *n*≥5; *P<0.05; **P<0.01.

OVX caused modest changes to the trabecular microarchitecture of cKO and WT mice compared with their equivalent sham operated control mice. Trabecular BV, trabecular number and trabecular thickness were all slightly diminished 4 weeks post-OVX in both genotypes and, although apparently more marked in cKO mice, these changes did not reach statistical significance (Figure 2A–E). A significant difference between genotypes was observed in the trabecular pattern factor (*P*<0.05; Figure 2G), indicating a more markedly disconnected trabecular structure in the Pdpn cKO mice than in WT mice following OVX (*P*<0.01; Figure 2G). This correlates closely with the structure model index which was also significantly increased in Pdpn cKO mice with OVX (*P*<0.01; Figure 2H). No effects of genotype or OVX were observed in trabecular BMD (Figure 2I). Cortical bone analysis indicated a significant decrease in BV/TV in WT mice with OVX (*P*<0.01), however no effect was observed in Pdpn cKO mice (Supplementary Figure S1). A modest decrease in cross-sectional thickness was also observed with OVX in WT mice, and this was significantly decreased in Pdpn cKO mice (*P*<0.05; Supplementary Figure S1). No significant differences were observed in other cortical bone parameters (Supplementary Figure S1).

### OVX induces alterations in osteocyte morphology in WT and Pdpn cKO mice

**Figure 3:**
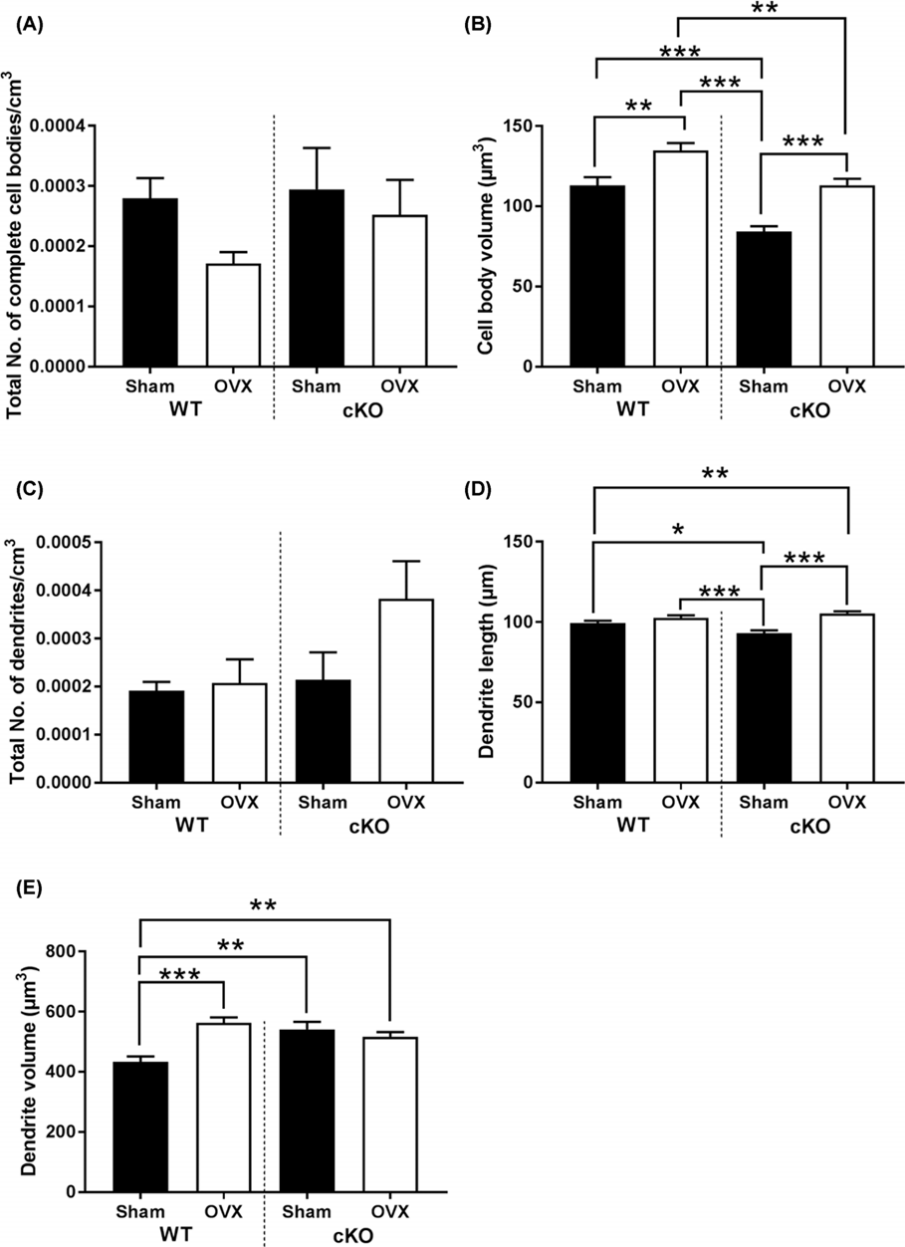
Osteocyte morphology in WT and Pdpn cKO mouse bone Quantification of osteocyte morphology parameters using phalloidin-Factin staining and 3D rendering. (**A**) Total number of complete cell bodies/cm^3^. (**B**) Cell body volume (μm^3^). (**C**) Total number of dendrites/cm^3^. (**D**) Dendrite length (μm). (**E**) Dendrite volume (μm^3^). Data are represented as mean ± SEM. *P<0.05, **P<0.01, ***P<0.001.

To further delineate the association between Pdpn and osteocyte formation with OVX, we next performed phalloidin staining of F-actin in WT and cKO cortical bone. Subsequent 3D rendering and quantitative analysis of osteocytes confirmed our previously published data: modest decreases in the total number of cell bodies (Figure 3A) and the total number of dendrites (Figure 3C), and significant decreases in cell body volume (*P*<0.001; Figure 3B), and dendrite length (*P*<0.05; Figure 3D) in sham-operated cKO mice compared with WT were observed. Interestingly, a significant increase in dendrite volume was noted in cKO mice compared with WT (*P*<0.05, Figure 3E). In WT mice, OVX significantly increased the cell body volume (*P*<0.01; Figure 3B) and dendrite volume (*P*<0.001; Figure 3E). Similar trends were seen in cKO mice with OVX, with significant increases in cell body volume (*P*<0.001; Figure 3B) and dendrite length (*P*<0.001; Figure 3D) observed.

### Gene expression of bone remodelling markers are unaffected by hypomorphic deletion of Pdpn

**Figure 4:**
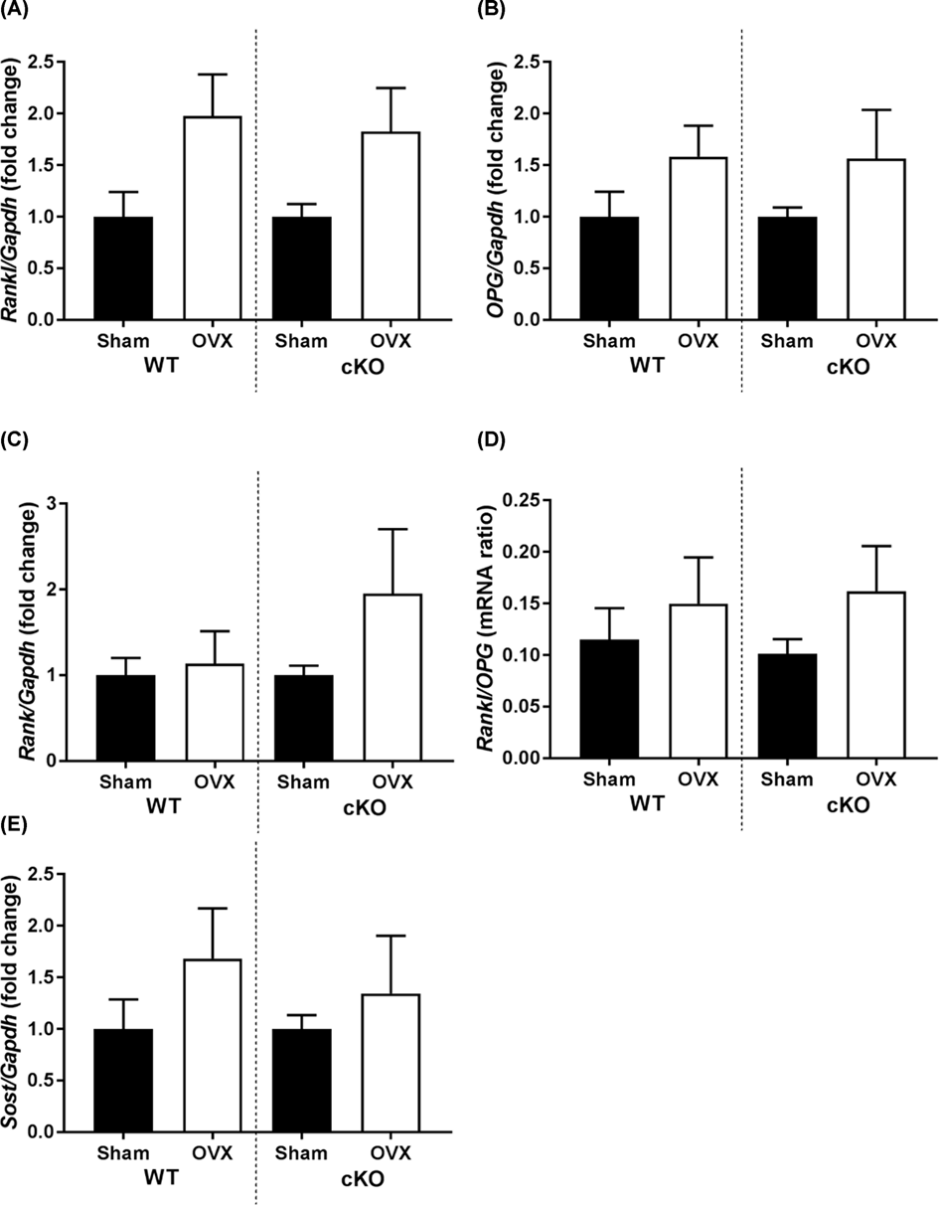
Gene expression in WT and Pdpn cKO mouse bone RT-qPCR analysis of (**A**) RANKL (Tnfsf11), (**B**) OPG (*Tnfrsf11b*), (**C**) RANK (*Tnfrsf11a*), (**D**) RANKL:OPG ratio, (**E**) sclerostin (*Sost*). Data are presented as mean ± S.E.M for *n*≥5.

The RANK/RANKL/osteoprotegrin (OPG) axis is major influence on bone remodelling and if the RANKL/OPG ratio becomes imbalanced then osteopaenia can result. Therefore we examined whether OVX-related changes in the expression of *Opg, Rankl* and *Rank* were affected by Pdpn deletion. Although trends were seen for increased *Rankl* and *Opg* expression in OVX mice, no statistically significant differences were observed between WT and cKO mice (Figure 4A,B). Similarly, no significant differences were observed in the expression of Rank (Figure 4C), and changes the *Rankl/Opg* ratio in response to OVX were similar in WT and cKO mice (Figure 4D). Sclerostin is a negative regulator of Wnt signalling and bone formation and is down-regulated in bones from OVX mice. However, in this present study we noted that although this increase did not reach significance, *Sost* expression was somewhat raised by OVX in both WT and cKO mice (Figure 4E).

### Deletion of Pdpn protected against increased osteoclast numbers with OVX

**Figure 5:**
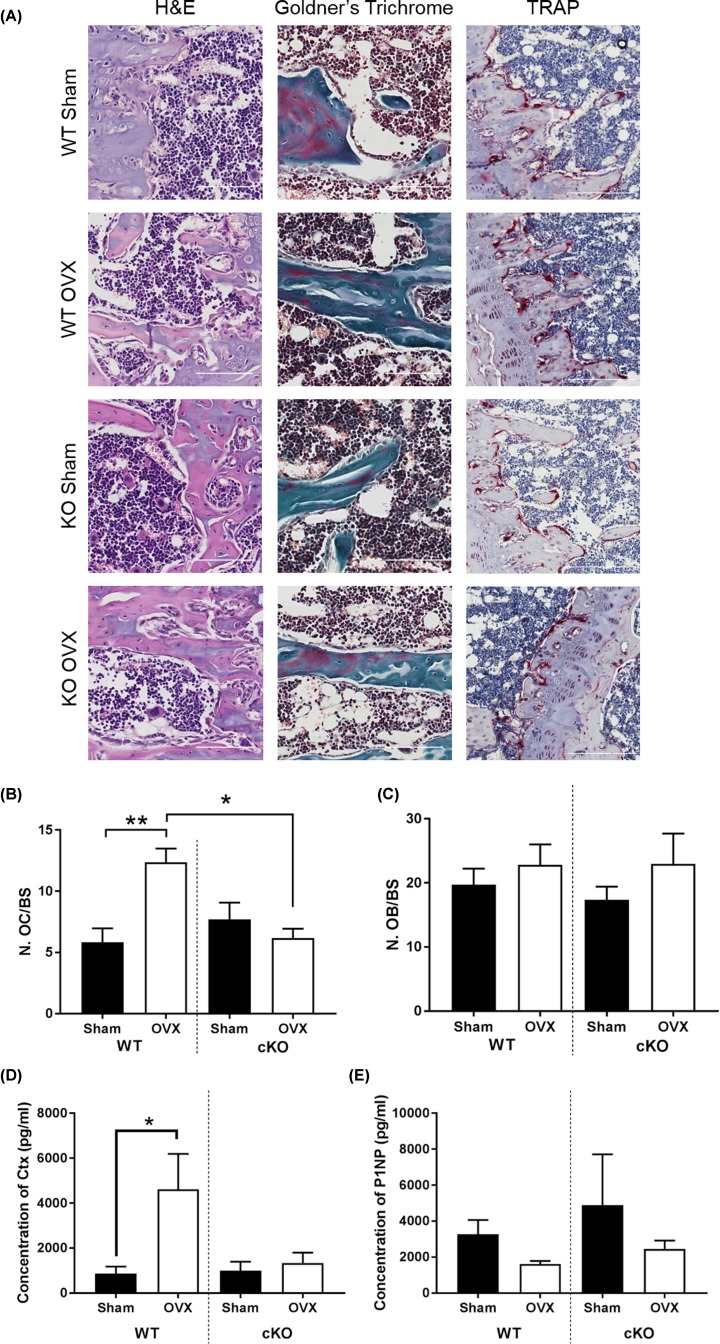
Effects of OVX on WT and Pdpn cKO mouse bone histology (**A**) Representative micrographs of WT and cKO tibiae following sham or OVX surgery, 20× magnification. Osteoclasts marked with black arrowheads. Histological analysis of cell counts in sham vs OVX bones. (**B**) Number of osteoclasts/bone surface. (**C**) Number of osteoblasts/bone surface. (**D**) Concentration of Ctx (marker of bone resorption). (**E**) Concentration of P1NP (bone formation). Data are presented as mean ± S.E.M for *n*≥5; *P<0.05; **P<0.01. Scale bars represent 200 µm.

Histological assessment of TRAP activity in WT mice following sham and OVX surgeries revealed an expected significant increase in osteoclast number per bone surface upon OVX surgery (*P*<0.01 in comparison with sham operated WT mice; Figure 5A,B). In contrast, the cKO mice appeared to be protected from the OVX-induced increase in osteoclast number, which instead was similar in OVX and sham-operated cKO mice (Figure 5A,B). A significant difference in osteoclast number per bone surface was also observed between WT and cKO OVX mice (*P*<0.05; Figure 5B). Serum levels of the resorption marker Ctx were also significantly increased in WT mice following OVX surgery but remained unchanged in cKO mice following OVX surgery (Figure 5D). In contrast, both osteoblast number (Figure 5C) and serum P1NP, a marker of bone formation, were unaffected by OVX surgery and were similar in WT and cKO OVX mice (Figure 5E).

## Discussion

This manuscript utilised our previously characterised *Pdpn* cKO mice to examine the effects of OVX-induced bone resorption in an attempt to decipher the *in vivo* role of Pdpn on the bone remodelling process. [14] Here we found that while the hypomorphic deletion of Pdpn had no marked effect on OVX-induced bone phenotype, at the time point studied it protected against OVX-related increases in osteoclast number and the Ctx marker of bone resorption.

The vast osteocyte dendritic network is critical for cell–cell communication in bone, maintaining cell viability and allowing the transfer of nutrients and waste products [10]. We and others have previously shown that Pdpn promotes osteocytogenesis and dendrite formation *in vitro* [11,12]. Similarly, we have previously shown that stabilisation of Pdpn protein, through inhibition of endogenous targeted proteasome activity, promotes dendrite formation *in vitro* [11]. By using the cre-LoxP system targeted to exon 3 of the *Pdpn* gene, we have recently generated bone-specific (OC-Cre) conditional knockdown mice. We revealed that while Pdpn deletion did not affect osteocyte differentiation *in vivo*, a vital role for Pdpn in the formation of full length dendritic processes was observed [14]. Here, our data confirm this and suggest that the hypomorphic deletion of Pdpn and the subsequent inadequate dendrite formation, quantified by phalloidin staining for F-actin, does not affect OVX-induced changes in the trabecular bone microarchitecture. Interestingly a modest increase in the total number of dendrites was observed in cKO mice upon OVX and this therefore may have influence on the ability to respond to skeletal load, but its relevance to the regulation of bone remodelling needs further investigation.

Numerous studies have reported the detrimental effects of oestrogen deficiency on osteocyte viability. Indeed, oestrogen maintains osteocyte viability and its depletion in OVX results in regional osteocyte apoptosis in animal models and in human bone biopsies [17–19]. Inhibition of osteocyte apoptosis through administration of the pan-caspase inhibitor QVD prevented increases in the osteoclastic resorption normally observed with OVX, thus suggesting a key role for osteocyte apoptosis in the initiation of endocortical remodelling in response to oestrogen deficiency [19]. Consistent with this, here we observed modest yet non-significant decreases in the total number of osteocyte cell bodies with OVX in WT mice. Therefore, future studies examining the viability of the osteocytes in our model would be of great interest. In particular, it would be interesting to see if our cKO mice exhibit decreased osteocyte apoptosis and hence are protected against OVX-induced increases in osteoclast activity.

Osteocytes play an integral role in maintaining bone homoeostasis by regulating bone modelling and remodelling through communicating with bone-resorbing osteoclasts via RANKL production. [3,4] Osteoclasts are specialised multinucleated cells that arise from bone marrow precursors and their differentiation is promoted by RANKL. Indeed the targeted disruption of RANKL in mice results in severe osteopetrosis [3,20]. OVX in mice stimulates bone resorption by increasing osteoclast formation and activity due to decreased oestrogen levels as is seen in women with postmenopausal osteoporosis. Here we observed expected increases in osteoclast number in WT mice subjected to OVX. However, Pdpn cKO mice exhibited a protection against OVX-induced increases in osteoclast number as well as activity, as indicated by TRAP activity and serum levels of the bone resorption marker Ctx respectively. This could be due, in part, to the known disruption to the osteocyte dendritic network previously reported in Pdpn cKO mice [14] and thus infer a decreased capacity to promote osteoclastogenesis through ineffective RANKL production by osteocytes lacking Pdpn. Our gene expression data suggested that no significant differences were observed in RANKL in either WT or Pdpn cKO mice, however this may be due to RANKL expression not being exclusive to osteocytes. Indeed RANKL can be produced by stromal cells, osteoblasts, T lymphocytes and B lymphocytes in bone [21]. Furthermore, it has been reported that osteoblasts are the major source of RANKL during oestrogen deficiency and thus suggest that the lack of changes in RANKL expression which we observe are independent of the osteocyte network [22]. A focus in future studies could be to further delineate this source of RANKL and could correlate changes in gene expression to that of protein expression for example through immunohistochemical labelling.

Compelling evidence for a role for osteocytes in regulating bone remodelling also comes from the discovery that osteocytes, deep in calcified bone, produce sclerostin, a Wnt inhibitor and potent negative modulator of bone formation [23–25]. Indeed, sclerostin has been implicated as a regulator of the differentiation from late osteoblast to pre-osteocyte, which antagonises Wnt signalling through binding to the Wnt co-receptors, low-density lipoprotein receptor-related proteins 5 and 6 (LRP5 and LRP6), thereby leading to β-catenin phosphorylation and subsequent degradation [26]. The therapeutic potential of targeting sclerostin has recently been exploited as an anabolic treatment for osteoporosis [27–30]. Here we observed trends toward increases in bone sclerostin mRNA expression in response to OVX, however, these trends failed to reach levels of statistical significance and no differences were observed between genotypes. This is consistent with our previous study in which we observed that the hypomorphic deletion of Pdpn exerted no effect on sclerostin levels *in vivo*, and failed to modify osteocyte number, shape or size in these mice [14].

In summary, our data confirm a role for *Pdpn* in OVX-induced bone remodelling and that an intact osteocyte network contributes to increases in osteoclastogenesis in OVX-induced bone loss, through mechanisms potentially independent of RANKL expression. This work will enable a greater understanding of role of osteocytes in bone loss induced by oestrogen deprivation.

## Supplementary Material

Supplementary Figure S1

## Abbreviations

BMD: bone mineral density
cKO: conditional knockout mice
OC: osteocalcin
OPG: osteoprotegrin
OVX: ovariectomy
PBS: phosphate buffered saline
Pdpn: Podoplanin
RANK: receptor activator of nuclear factor κ B
RANKL: RANK-ligand
TRAP: tartrate-resistant acid phosphatase
WT: wild-type
