# Supplementary material for: Conditional deletion of E11/Podoplanin in bone protects against ovariectomy-induced increases in osteoclast formation and activity
Source: Biosci Rep. 2020 Jan 10;40(1):BSR20190329. doi: 10.1042/BSR20190329 (PMC6954370; doi:10.1042/BSR20190329)
Supplement: Supplementary Figure S1 [file BSR-2019-0329_supp.pdf]

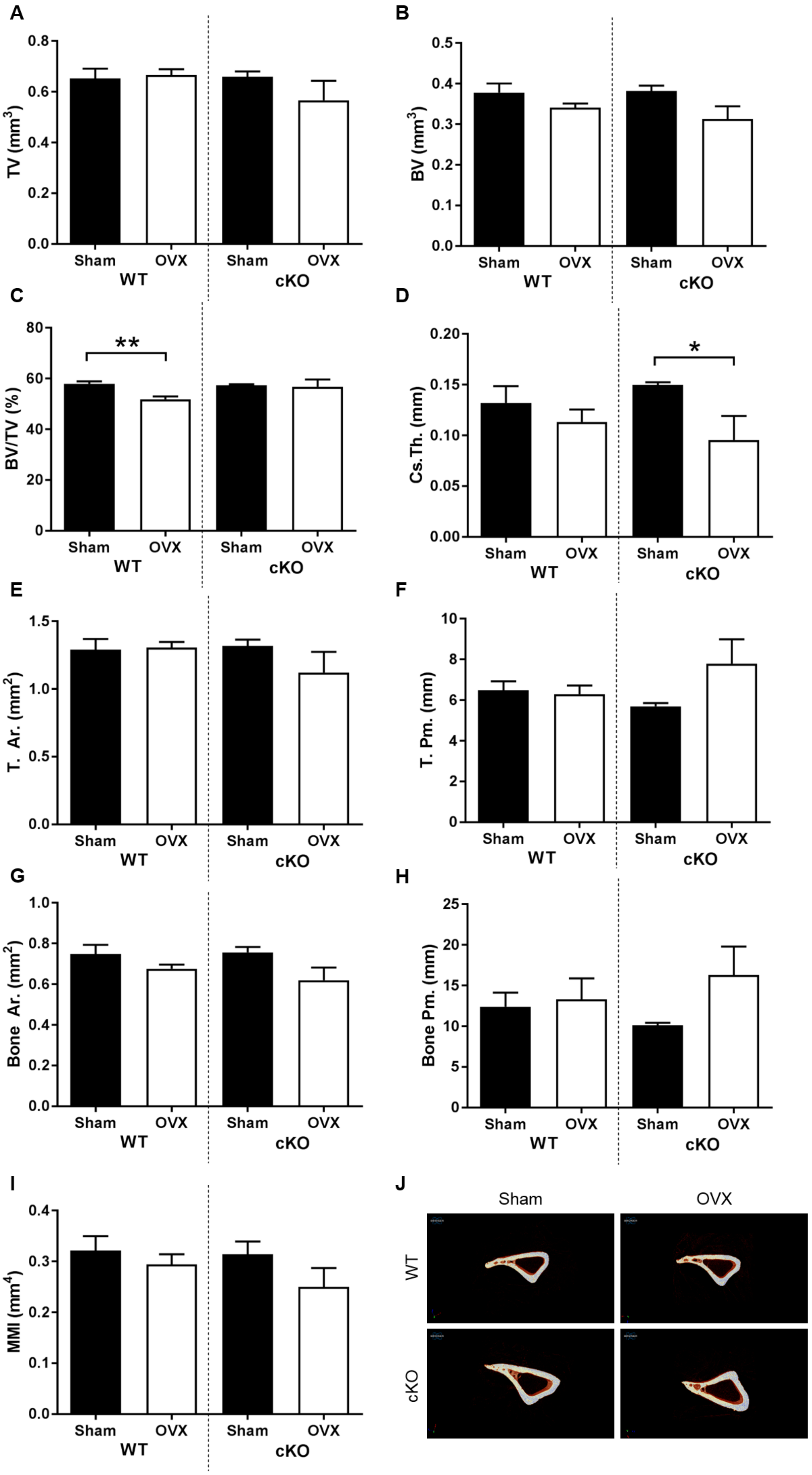

A
TV (mm3)
0.8
0.6
0.4
0.2
0.0
Sham
OVX
Sham
OVX
WT
cKO
B
BV (mm3)
0.5
0.4
0.3
0.2
0.1
0.0
C
BV/TV (%)
80
60
40
20
0
**
D
Cs.Th. (mm)
0.20
0.15
0.10
0.05
0.00
*
E
T. Ar. (mm2)
1.5
1.0
0.5
0.0
F
T. Pm. (mm)
10
8
6
4
2
0
G
Bone Ar. (mm2)
1.0
0.8
0.6
0.4
0.2
0.0
H
Bone Pm. (mm)
25
20
15
10
5
0
I
MMI (mm4)
0.4
0.3
0.2
0.1
0.0
J
Sham
OVX
WT
cKO

Suppl. Fig. 1. Effects of OVX on WT and PdpncKO mouse cortical bone geometry. Micro-CT analysis of tibia cortical bone geometry in WT and PdpncKO mice in response to OVX **(A)** TV (corticaltissue volume; mm^3)**(B)** BV (cortical bone volume; mm^3)**(C)** BV/TV (bone volume/tissue volume; %)**(D)** Cs. Th. (Cross-sectional thickness; mm) **(E)** T. Ar. (Mean total cross-sectional tissue area; mm^2) **(F)** T. Pm. (Mean total cross-sectional tissue perimeter; mm) **(G)** Bone Ar. (Mean total cross-sectional bone area; mm^2) **(H)** Bone Pm. (Mean total cross-sectional bone perimeter; mm) **(I)** MMI (Mean polar moment of inertia; mm^4).**(J)** Representative images. Data are presented as mean ± S.E.M for $n \geq 5$; $^{**}p<0.01$.
